# Diffuse subarachnoid hemorrhage following ventriculo-peritoneal shunt insertion for acute obstructive hydrocephalus from large glomus jugulare tumor: case report

**DOI:** 10.3389/fsurg.2024.1353400

**Published:** 2024-04-05

**Authors:** Mestet Yibeltal Shiferaw, Tsegazeab Laeke Teklemariam, Eyob Zenebe Wondimagegnewu, Dejen Tekiea Gebrewahd, Ermias Fikru Yesuf, Bereket Hailu Mekuria, Sebboona Baisa Abelti

**Affiliations:** ^1^ Department of surgery, Neurosurgery Unit, Debre Tabor University, Debre Tabor, Ethiopia; ^2^ Department of surgery, Neurosurgery Division, Addis Ababa University, Addis Ababa, Ethiopia

**Keywords:** endovascular embolization, glomus jugulare tumors, hydrocephalus, subarachnoid hemorrhage, vascular malformation, ventriculoperitoneal shunt insertion

## Abstract

**Background:**

Glomus jugulare tumors (GJTs) are rare intra-cranial tumors. Commonly, these lesions present with cranial nerve palsies, headaches, and hydrocephalus. Rarely, GJTs present with spontaneous subarachnoid hemorrhage. However, there has never been a report of diffuse subarachnoid hemorrhage following ventriculoperitoneal shunt insertion in a patient who developed hydrocephalus secondary to any brain tumor in general or glomus jugulare tumors in particular.

**Observation:**

The authors presented an extremely rare complication of diffuse subarachnoid hemorrhage following the insertion of a ventriculoperitoneal shunt (VPS) in a 61-year-old female patient who was diagnosed to have both clinical and radiologic features of acute obstructive hydrocephalus secondary to a highly vascular huge glomus jugulare tumor.

**Conclusion:**

Subarachnoid hemorrhage following ventriculoperitoneal shunt insertion for hydrocephalus caused by a mass lesion is an extremely rare complication. Preoperative CT angiography should be strongly considered to look for the associated vascular malformations in extremely vascularized mass lesions. Given the not ubiquitous availability of all therapeutic options for GJTs, especially in low and middle income settings contributes for the poor outcome of GJTs and it fosters a global neurosurgery agenda.

## Introduction

Glomus jugulare tumors (GJT) are rare lesions with an annual incidence of approximately 1 in 1 million people per year (1–3). These tumors affect females more frequently than males and commonly occur in the sixth to seventh decade of life (4). This tumor grows so slowly that it doubles itself in 4.2 years (5). The clinical presentation of glomus jugulare tumors is variable, and the specific neurologic symptoms depend on the region affected by the tumor (6–10).

Most paragangliomas located below the neck are functional unlike <4% to head and neck paragangliomas (11–13). If there is suspicion of secretory glomus jugulare tumors on clinical examination, confirmatory laboratory tests like 24 h urine metanephrines and fractionated catecholamines, as well as plasma metanephrines, can be done.

While placement of a ventriculoperitoneal shunt is associated with common complications like shunt infection, malfunction, and subdural hygroma and hematoma (SDHs) as a result of cerebrospinal fluid (CSF) overflow (14–18), Here, we present diffuse subarachnoid hemorrhage as an exceedingly rare complication of ventriculoperitoneal shunt (VPS) in a patient who was diagnosed to have both clinical and radiologic features of acute obstructive hydrocephalus secondary to a highly vascular, huge glomus jugulare tumor.

## Illustrative Case

A 61-year-old woman who was referred from a private hospital to us presented with a decreased level of consciousness for 4 days and worsening vomiting and headache. She started to have a global headache, repeated episodes of vomiting, and gait disturbances lasting 4 months. In addition, she also had decreased hearing and tinnitus for 4 years and had intermittent chocking during swallowing of 1 year duration. She received mannitol and high-dose dexamethasone in a private hospital for the decreased level of consciousness and intractable vomiting. Otherwise, she had no known chronic medical illnesses and no prior history of surgery.

On a physical exam, she was acutely chronically ill-looking. She had normal vital signs. The neurologic exam showed a Glasgow Coma Scale of 14/15 (she was confused). Pupils were mid-sized and reactive bilaterally. Cranial nerves VII, VIII, IX, and X were affected on the right side. Cerebellar signs were also positive. She moves all her extremities and has no other neurologic deficit.

Blood workups, including a complete blood count, liver and renal function tests, and serum electrolytes, were normal.

**Figure 1:**
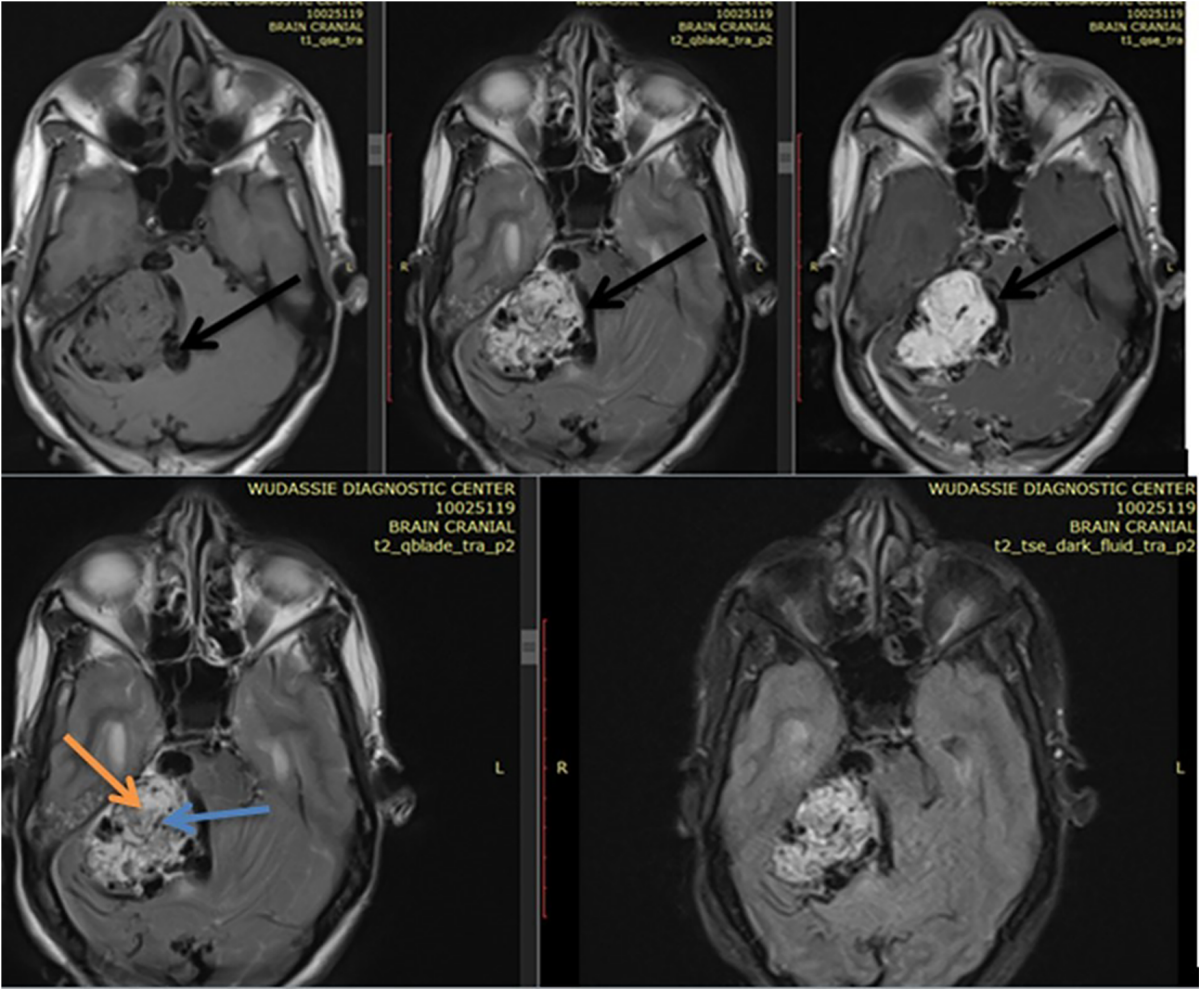
Shows a right side 5 by 5 cm large jugular foramen mass predominantly iso intense on T1 & hyper intense on T2 with extensive multiple & large size intra & peritumoral flow voids as it appears hypointense on both T1 & T2 MRI (see dark arrows of Figure 1). The dark arrow on postcontrast axial MRI in figure shows the “drop out” phenomenon. The tumor also had “salt-and-pepper” pattern (the orange & blue arrow shows the salt & pepper respectively as seen in Figure 1).

**Figure 2:**
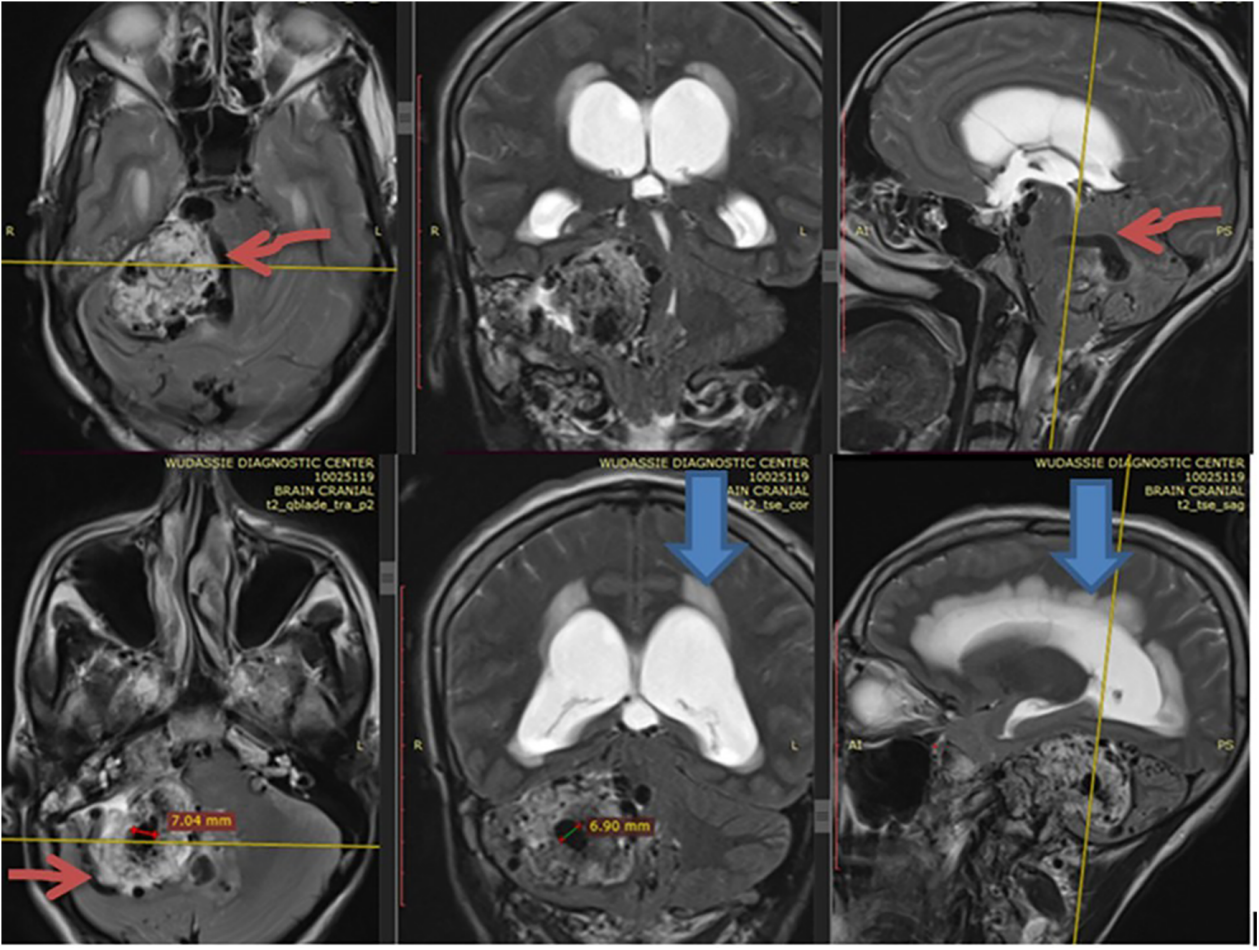
Shows with extensive multiple & large size intra & peritumoral flow voids (depicted by the dark red) as it appears hypointense on T2 MRI in axial, coronal and sagittal section. The blue arrow also shows acute hydrocephalus.

**Figure 3:**
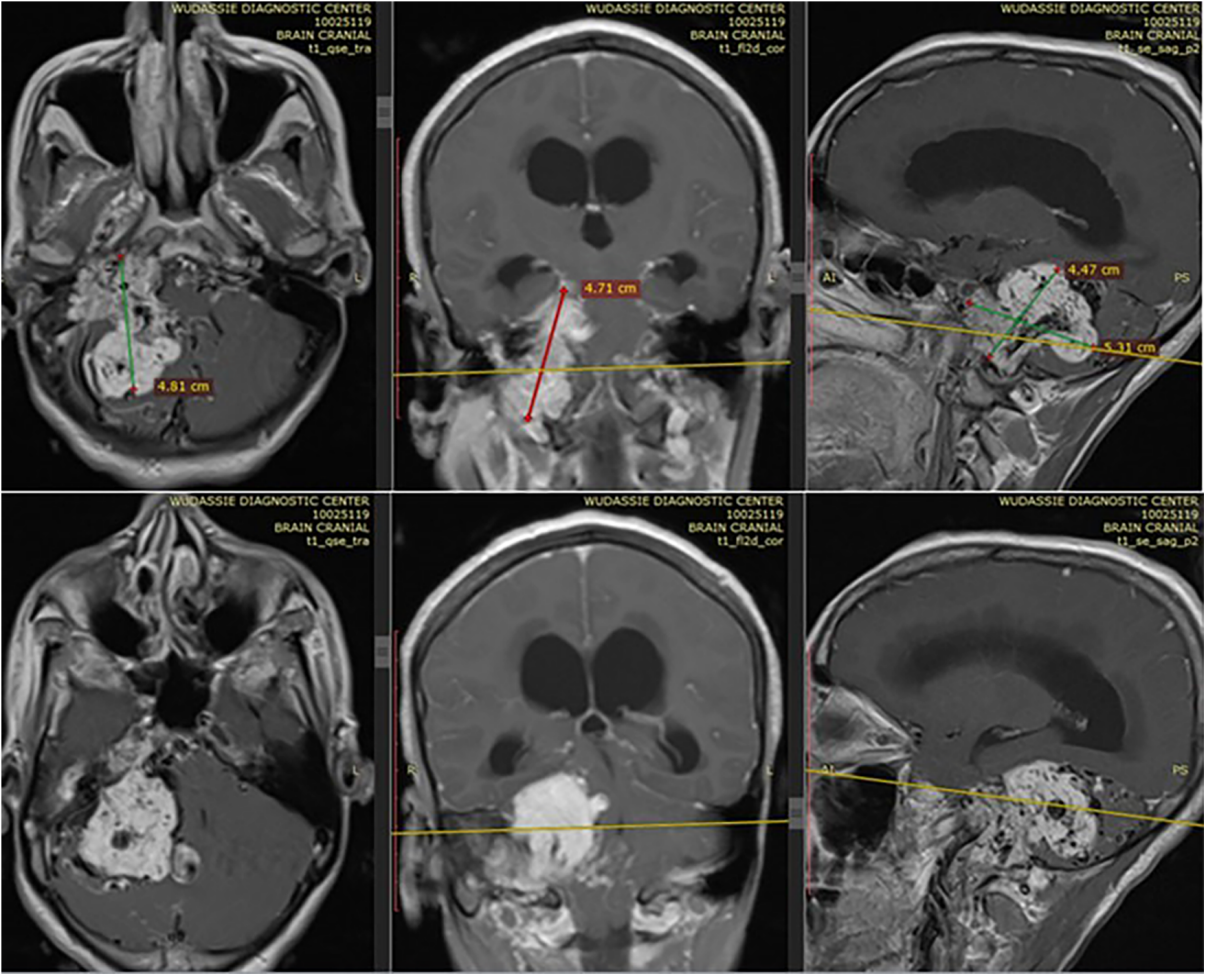
Shows the avid post contrast enhancement in different planes of sections. It also shows the extension of tumor to middle ear, mastoid air cells and part of petrous bone.

She came with a brain MRI, and it showed a 5 by 5 cm large jugular foramen mass that has a large intracranial and small middle ear extension. The lesion was predominantly iso- and hyper-intense on T1 and T2, respectively, with extensive, multiple, and large intra- and peritumoral flow voids, as it appeared hypointense on both T1 and T2 MRIs (see dark and dark red arrows in Figures 1, 2, respectively). The tumor also had a “salt-and-pepper” pattern (the orange and blue arrows show the salt and pepper, respectively, as seen in Figure 1) due to multiple areas of flow void from high vascularity and hyperintense foci produced by slow flow or subacute hemorrhage, respectively, on both T1 and T2 images. The tumor had avid post-contrast enhancement (see Figure 3), and the multiple intra- and peritumoral flow voids still appear hypo-intense even on a post-contrast MRI, suggesting a phenomenon called “drop out” (as seen by the dark arrows in Figure 1, including on the post-contrast image), which is commonly found in glomus jugulare tumors. Acute obstructive hydrocephalus, as evidenced by tri-ventriclar enlargement and cerebrospinal fluid (CSF) transependymal transudation, was also noted, as seen by the blue arrow in Figure 2.

**Figure 4:**
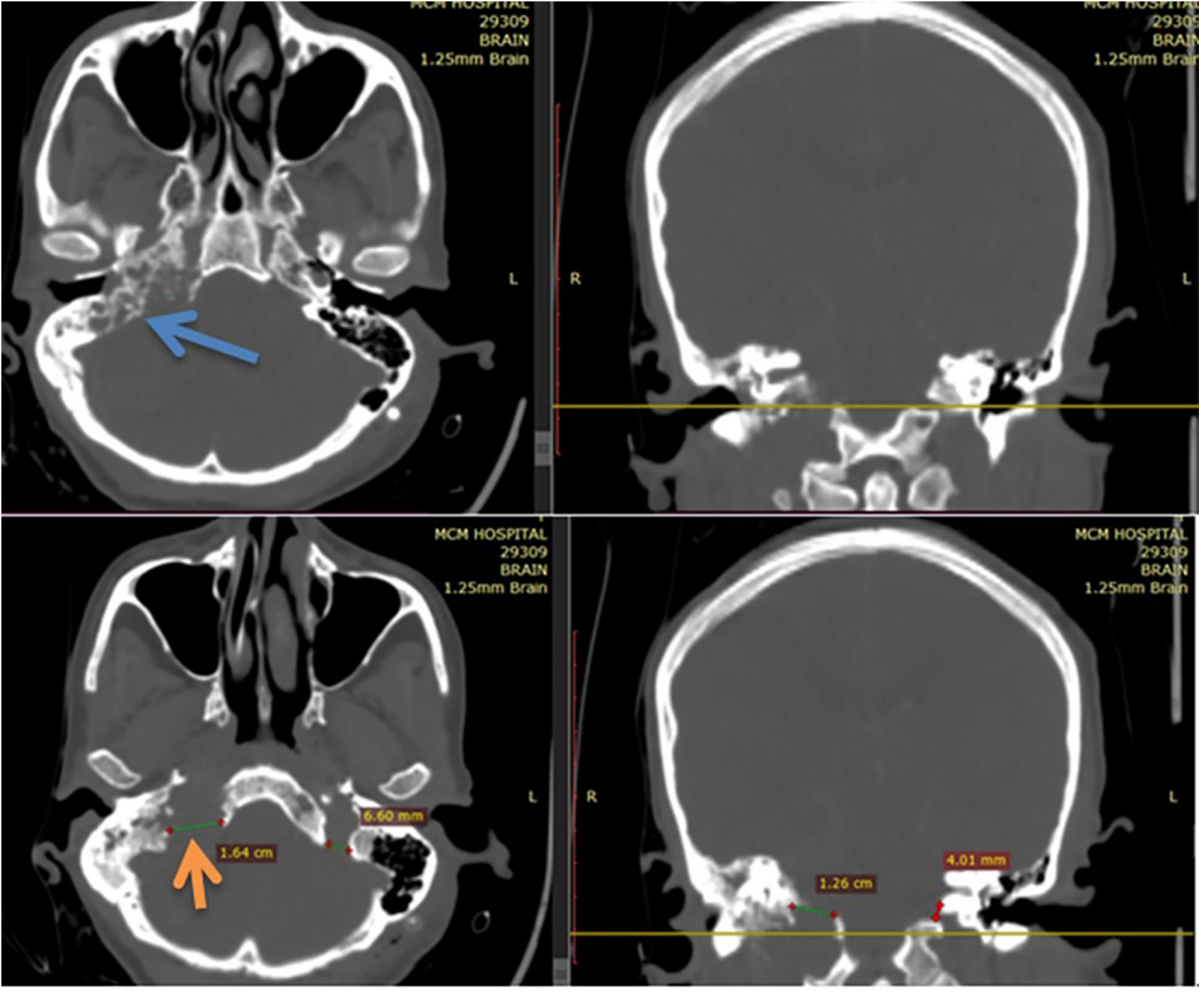
Bone window of CT scan showing an extremely widened right side jugular foramen (depicted by the orange arrow of Figure 4) with a characteristic “moth-eaten” pattern (depicted by blue arrow of Figure 4) of destruction of petrous part of temporal bone.

The bone window of the CT scan showed an extremely widened right side jugular foramen (depicted by the orange arrow in Figure 4) with a characteristic “moth-eaten” pattern (see the blue arrow in Figure 4) of destruction of the petrous part of the temporal bone.

Altogether, an imaging diagnosis of acute obstructive hydrocephalus (AOHCP) from the right side of a large, highly vascularized glomus jugulare tumor was made. CT angiography was not done as her neurologic condition from the acute hydrocephalus warranted emergency treatment.

## Surgery

Written informed consent was obtained, and a left-side keen's point VPS was inserted. Upon insertion of the proximal catheter into the ventricle, clear CSF came under high pressure, but only 3 cc of CSF was taken for analysis. The patient had delayed awakening due to an opioid overdose (evidenced by pinpointed pupils bilaterally and the rapid awakening following the administration of naloxone). She was then extubated on the table and transferred to the intensive care unit for close observation, though she had stable immediate post-operative neurologic status.

## Postoperative course

**Figure 5:**
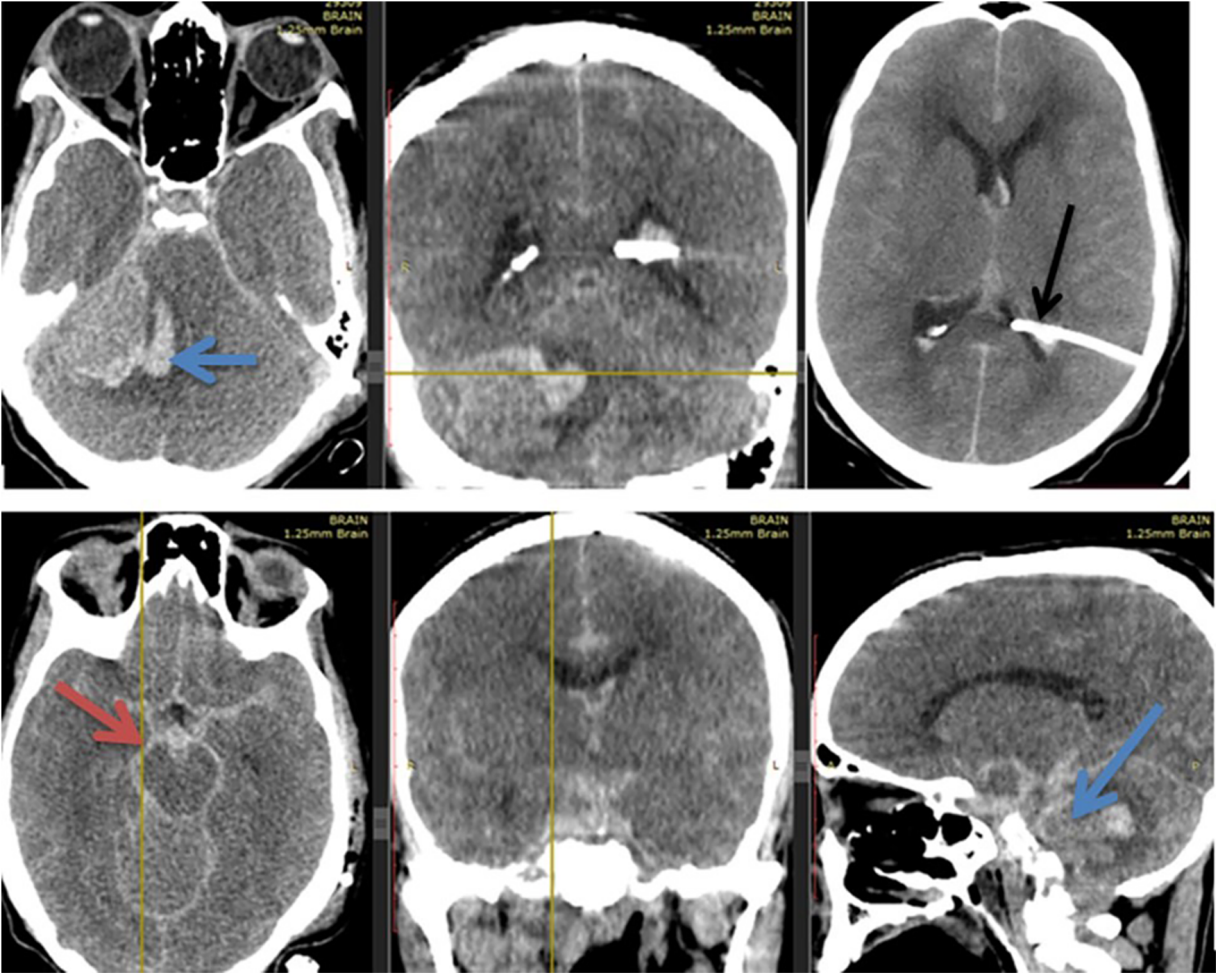
Shows the control CT scan of the patient immediately after neurologic deterioration and showed diffuse basal cistern thick subarachnoid hemorrhage with effaced basal cisterns (marked by the dark red arrow), minimal intraventricular hemorrhage and intra & peritumoral bleeding (as depicted by the dark blue arrow). The proximal shunt tip catheter was inside the left atrium (marked by the dark arrow). There was no malposition & signs of over drainage.

From the immediate period to 24 h after the surgery, the patient was communicating and had a GCS of 14–15/15. Her headache and vomiting improved. She also didn't have a post-OP neurologic deficit. After 24 h of stay in the ICU, she suddenly had deterioration in a GCS from 14/15 to 3/15. Emergency intubation was then done, and an emergency control CT scan was immediately obtained, which showed diffuse basal cistern thick subarachnoid hemorrhage with effaced basal cisterns (marked by the dark red arrow in Figure 5), minimal intraventricular hemorrhage, and intra- and peritumoral bleeding (as depicted by the dark blue arrow in Figure 5). The proximal shunt tip catheter was inside the left atrium (marked by the dark arrow in Figure 5). There was no malposition or signs of over-drainage. For this, medical treatment for subarachnoid hemorrhage of aneurysmal origin was initiated. Her blood pressure was not above 160/90 mmHg, and she didn't require labetalol. Despite these treatments, her GCS remained 2T, and brainstem reflexes became absent and passed away after 10 h of her deterioration, with the possible cause of death being post-shunt insertion-related bleeding of the highly vascular tumor causing severe diffuse subarachnoid hemorrhage and its complications.

## Discussion

### Observation

GJTs are rare, slowly growing, locally destructive, highly vascular tumors, accounting for approximately 0.6% of head and neck tumors. GJTs are the most common jugular foramen (JF) tumors that arise from paraganglion cells in the area of the jugular bulb (1–3, 19).

Commonly, patients present with tinnitus, hypoacusis, headache, vertigo, and multiple cranial nerve palsy, with possible involvement of the 5th to 12th cranial nerves, though the whole clinical picture of patient presentation is a function of the region affected by the tumor. These symptoms are all consistent with the presenting symptoms of our patient. The symptom related to hydrocephalus in our patient is partly explained by an extremely large tumor with a mass effect due to delayed presentation and is extremely rare.

Radiologically, the hallmark of GJTs that will help differentiate it from other tympanic masses (e.g., cholesteatoma) is its strong enhancement with contrast, which is indicative of hypervascularity. GJTs give an MR appearance known as the “salt-and-pepper” pattern on both T1 and T2 images. The “pepper” component is caused by multiple areas of flow void due to high vascularity, interspersed with the “salt” component, which is caused by hyperintense foci produced by slow flow or sub-acute hemorrhage on both T1 and T2 images (20–26).

GJTs had avid post-contrast enhancement, and the multiple intra- and peri-tumoral flow voids still appear hypo-intense even on a post-contrast MRI, suggesting a phenomenon called “drop out”. GJTs are the only neoplasms of the skull base to exhibit the “dropout” phenomenon after intravenous injection of gadolinium. This drop phenomenon is a result of the early disappearance of blushing and the early washout of contrast due to high flow. Angiography helps rule out vascular malformations and defines the feeding arteries to the tumors. It is especially mandatory when one plan to administer preoperative endovascular embolization and GJTs generally have a shorter blush time than meningioma.

A thin-slice bone-windowed CT shows a smoothly scalloped, well-corticated enlargement of the JF in the case of lower cranial nerves, in contrast to the moth-eaten pattern that is seen with GJTs or the irregular osteolytic destructive pattern of metastases or lymphomas (25–27).

Surgery is the mainstay of treatment for GJTs, as it is the only treatment option that can offer immediate and complete tumor elimination, though surgical treatment is challenging. The technical difficulty of surgery emanates from the highly vascular nature of this tumor, poorly accessible skull base regions, and the tendency of this tumor to have multi-compartment involvement and engulfment of critical neurovascular structures (7–9, 28–32). Hence, radiotherapy with the goal of disease control or growth inhibition rather than tumor elimination (7–10, 33–37), and preoperative endovascular embolization, which is vital in decreasing blood loss during surgical resection, is helpful adjuncts in the treatment of these tumors.

Accordingly, there is an increasing evidence that the use of gamma knife radiosurgery (GKR) may play a relevant role as a therapeutic option in these tumors, particularly strategies for endovascular embolization plus fractionated GKR (Ganau 2014) and availability of radioenhancers (Ganau, 2015) can expand even further its indication for patients harboring large GJTs. Of note, Gerosa et al. demonstrated that GKR was safe and effective in 20 GJTs patients, mostly classified as Glasscock-Jackson Grade IV or Fisch Stage D1 (average tumor volume: 7.03 cm, range: 1.5–13.4 cm) who were treated with a mean marginal dose of 17.3 Gy (range, 13–24 Gy). In their series, an improvement of cranial nerve function was observed in 25% of cases, a decrease in tumor size was observed in 40% (Gerosa, 2006). Keeping in mind that GJTs are estimated to double in size every 4 years, a multispecialistic treatment, with consideration for surgical, endovascular, and radiosurgical options, is essential to achieve an effective tumor growth control with negligible incidence of untoward sequelae. Reflecting on the disparities between high income countries and low and middle income countries is also essential given the not ubiquitous availability of all the options above, which make the poor outcome of GJTs that fosters a global neurosurgery agenda (38–40).

An uncommon presentation that complicates the treatment of patients with glomus jugulare is the presence of AOHCP, especially when clinical and imaging features of acute OHCP are seen in our patient. While straight-forward treatment of AOHCP caused by a large GJT by making use of CSF diversion methods can be done, the choice of CSF diversions can be different across different institutions. These strategies include VPS placement, endoscopic third ventriculostomy, and external ventricular drainage.

The extensive list of potential complications caused by shunts and 93% (57/61 patients) shunt independence following posterior fossa complete tumor excision reaches 100% and 83% in adults and children, respectively, with the use of external ventricular drain/subcutaneous reservoir/placement along with short-course perioperative steroid administration, and this speaks strongly against the routine use of shunt (41).

Taking the Frankfurt Grading System of adults for predicting the need for postoperative CSF diversion following posterior fossa tumor (42) into account besides the acute clinical and radiologic signs of acute hydrocephalus, emergency-based CSF diversion was decided. Of the CSF diversion procedures, ventriculoperitoneal shunting or external ventricular drains were too competitive. But because the tumor was extremely vascular, going for tumor excision without preoperative tumor feeder vessel embolization was not planned. That means that inserting an external ventricular drain was not a favorable option for our setting, where we didn't have endovascular embolization, as it would have a high risk of infection until the patient was referred abroad for the endovascular embolization and/or definitive tumor excision. Hence, ventriculoperitoneal shunt insertion was more favorable than external ventricular drain, and this is why our patient underwent medium-pressure VPS placement.

Unfortunately, the patient developed rapid neurologic deterioration after 1 day of the VPS insertion. Follow up CT scan of brain showed a postoperative diffuse subarachnoid hemorrhage of major basal cisterns and intra-tumoral bleeding with moderate ventricular size reduction. This diffuse subarachnoid hemorrhage in literature following VPS insertion is extremely rare and is never reported following OHCP from GJTs to the best of authors' knowledge. However, there are only 2 case reports of patients with glomus jugulare presenting with subarachnoid hemorrhage. The hemorrhages in these two case reports were spontaneous and without surgery but the patients were found to have high blood pressure (43, 44), in exact contrast to our patient, who developed post-VPS insertion and had normal range blood pressure throughout her clinical course. Common to our case and these case reports was that the tumor was highly vascular, even if the degree of vascularity in our patient was far greater. An MRI with contrast of our patient and CT angiography done on the case reports revealed no aneurysm or arteriovenous malformation. CT angiography (CTA) for a detailed vascular study to rule out aneurysms and vascular malformations was not done in our patient as her acute hydrocephalus warranted urgent treatment.

What caused the subarachnoid hemorrhage in our patient was probably the extremely tortuous and dilated vessels inside and around the tumor. The high degree of engorgement might also contribute to the thinning of the vessels' walls, partly contributing to the hemorrhage even following the minor hydrodynamic changes expected following the shunt placement. But, it was clear that the hemorrhage in our patient was neither from over drainage of the shunt (as the control CT of our patient never showed signs of over drainage) nor from iatrogenic hemorrhage due to shunt tip misplacement, as the tip of the shunt is inside the ventricle, as illustrated on the control CT scan. Hence, the fragility of these vessels in and around the tumor was likely to be the cause of hemorrhage, as the most common bleeding-associated shunt placement over drainage is subdural collections or intracerebral hemorrhage (15–18), and post-VPS insertion SAH is extremely rare. The treatment of acute hydrocephalus in this patient should have had preoperative CTA (for detailed vascular studies), and preoperative endovascular embolization before VPS insertion would have been a more convenient strategy in treating hydrocephalus caused by extremely vascular tumors like ours, even if there is no evidence supporting this to the authors' best knowledge.

This case report has its own strengths and limitations. Accordingly, swift diagnosis and doing ventriculoperitoneal shunting for the acute hydrocephalus due to the large tumor was considerably good therapeutic approach followed, as far as the our setup was concerned. In addition, the choice of ventriculoperitoneal shunting for cerebrospinal fluid diversion was appropriate as definitive surgical resection was not planned in our setup due to the absence of endovascular and stereotactic radiotherapy complements. Meticulous execution of shunt insertion was also done to avoid over drainage associated bleeding and upward herniations. Similarly, appropriate postoperative patient follow-up in the intensive care unit was among the strengths. However, the attending team of physicians was obsessed with the acute hydrocephalus more and was not very observant of the need of getting CT angiography to rule out the possibility of vascular malformation or aneurysms around the highly vascularized tumor. Similarly, placing external ventricular drain was out of favor in our case though it could drain cerebrospinal fluid in a more controlled manner than ventriculoperitoneal shunting as definitive tumor resection was not planned with the gadgets we have in our setup.

## Conclusion

Subarachnoid hemorrhage following ventriculoperitoneal shunt insertion for hydrocephalus caused by a mass lesion is an extremely rare complication. Preoperative CT angiography is recommended to look for associated vascular malformations in extremely vascularized mass lesions. Given the not ubiquitous availability of all therapeutic options for GJTs, especially in low and middle income settings contributes for the poor outcome of GJTs and it fosters a global neurosurgery agenda.

## Data Availability

The datasets presented in this article are not readily available because of ethical and privacy restrictions. Requests to access the datasets should be directed to the corresponding author.
